# Drug Release Kinetics of Electrospun PHB Meshes

**DOI:** 10.3390/ma12121924

**Published:** 2019-06-14

**Authors:** Vojtech Kundrat, Nicole Cernekova, Adriana Kovalcik, Vojtech Enev, Ivana Marova

**Affiliations:** 1 Department of Food Chemistry and Biotechnology, Faculty of Chemistry, Brno University of Technology, Purkynova 118, 612 00 Brno, Czech Republic; kundrat@fch.vut.cz (V.K.); xccernekova@fch.vut.cz (N.C.); marova@fch.vut.cz (I.M.); 2 Department of Physical and Applied Chemistry, Faculty of Chemistry, Brno University of Technology, Purkynova 118, 612 00 Brno, Czech Republic; enev@fch.vut.cz

**Keywords:** biomaterials, electrospinning, drug release kinetics, levofloxacin, morphology, poly(3-hydroxybutyrate), scaffolds

## Abstract

Microbial poly(3-hydroxybutyrate) (PHB) has several advantages including its biocompatibility and ability to degrade in vivo and in vitro without toxic substances. This paper investigates the feasibility of electrospun PHB meshes serving as drug delivery systems. The morphology of the electrospun samples was modified by varying the concentration of PHB in solution and the solvent composition. Scanning electron microscopy of the electrospun PHB scaffolds revealed the formation of different morphologies including porous, filamentous/beaded and fiber structures. Levofloxacin was used as the model drug for incorporation into PHB electrospun meshes. The entrapment efficiency was found to be dependent on the viscosity of the PHB solution used for electrospinning and ranged from 14.4–81.8%. The incorporation of levofloxacin in electrospun meshes was confirmed by Fourier-transform infrared spectroscopy and UV-VIS spectroscopy. The effect of the morphology of the electrospun meshes on the levofloxacin release profile was screened in vitro in phosphate-buffered saline solution. Depending upon the morphology, the electrospun meshes released about 14–20% of levofloxacin during the first 24 h. The percentage of drug released after 13 days increased up to 32.4% and was similar for all tested morphologies. The antimicrobial efficiency of all tested samples independent of the morphology, was confirmed by agar diffusion testing.

## 1. Introduction

The goal of controlled drug delivery is to deliver a drug with a specific release profile to the targeted cells, tissues or organs [1]. In the area of controlled drug delivery, many release systems based on synthetic or natural polymers have been reported. Since 1950, three generation of drug delivery systems (DDS) have been developed. The first-generation DDS mainly included modifications of the drug release mechanisms, such as dissolution-controlled, diffusion-controlled, osmosis-controlled and ion-exchange-controlled mechanisms in oral and transdermal delivery. The advances in these first -generation DDS have been described in several excellent reviews [2,3,4]. The second-generation DDS, also known as smart drug delivery systems, were focused on zero-order release and introduced smart delivery systems encompassing nanoparticles, smart polymers and hydrogels, peptide and protein delivery. A large number of existing studies in the literature have reviewed the smart DDS and their clinical potential [5,6,7,8]. The third-generation DDS is still under development and involves modulated delivery systems, which would overcome the biological and physicochemical barriers [9,10]. Recently, a number of DDS have been developed based on biodegradable polymers, including polylactides [11,12], poly(lactide-co-glycolide) [13,14], poly(ε-caprolactone) [15], polyhydroxyalkanoates [16,17], chitosan, dextran, alginic acid and hyaluronic acid [18,19]. The use of the polymer matrix based on biodegradable polymer brings many advantages but the most important one is their (bio)degradation into oligomers and monomers, which can be metabolized through normal physiological pathways [20,21].

Polyhydroxyalkanoates (PHA) are biopolyesters of 3-hydroxyalkanoic acids synthesized and accumulated in bacterial cells as intracellular carbon and energy storage materials [22]. A broad range of PHAs with different physicomechanical properties can be biosynthesized using different substrates, co-substrates and microorganisms [23]. One of the disadvantages is their much higher cost compared to traditional petroleum-based polymers. The main reason for this is that the microbial syntheses of PHA require upstream and downstream processing, which are time, material and equipment demanding processes. Due to their high biocompatibility, non-toxicity and degradability in vivo, PHA polymers display high potential for application in drug delivery and tissue engineering [24]. The most extensively studied are poly(3-hydroxybutyrate) (PHB) and poly(3-hydroxybutyrate-*co*-valerate) (PHBHV), mainly due to their availability on the market. Both PHB and PHBHV are semi-crystalline polymers with molecular weights ranging from 200 to 3000–8000 kg·mol^−1^ [25]. Their degradation in body fluids occurs mainly through surface and bulk erosion combined with hydrolytic chain scission. This way of degradation is an attractive release mechanism for DDS [26]. Degradation of PHA materials in vitro depends on their chemical structure, molecular weight, crystallinity, sample dimensions, pH and temperature of the hydrolytic medium [27,28]. It was found that in vivo as well as in vitro degradation of PHA is much slower compared to other biopolyesters, e.g., polylactides (PLA). Gogolewski et al. reported that after six months of subcutaneous implantation using injection molded PHA samples in mice, only about 15–43% of the material was degraded. Polylactide-based materials under the same experimental conditions reached much higher degradation values in the range of 56–99% of the materials [29].

Numerous processing approaches are used for the preparation of the controllable drug delivery systems based on biodegradable polymers, including encapsulation, nanotechnology and electrospinning [30]. Electrospinning is a processing method for the preparation of fibrous polymer mats with different morphology and the fibers can be coated onto any surface, which offers great potential in tissue engineering [31]. Due to the slow hydrolysis rate, PHB electrospun fibers are preferable in tissue engineering and disease diagnosis but only with limitations in the DDS [32,33,34]. Therefore, various strategies have been used to modify drug release profiles from PHB. One of the ways of doing this is the blending of PHB with a polymer that has a shorter degradation time, for example with polyethylene oxide [35], and polylactides [31,36]. The next methodology to accelerate the degradation is the use of PHA with a chemical structure different to PHB, for example, the amorphous poly(3-hydroxybutyrate-*co*-4-hydroxybutyrate), where the presence of 4-hydroxybutyrate units in polyester accelerates the rate of hydrolytic degradation [37,38]. Another way of accelerating the degradation in vivo or in vitro is the modification of the surface morphology of PHB electrospun fibers loaded with the drug. The morphology and porosity of electrospun nonwoven meshes can be adjusted by many parameters such as polymer type (molecular weight, molecular weight distribution and architecture), solvent properties (solution concentration, viscosity, conductivity and surface tension), process parameters (electric potential, flow rate and concentration, deposition distance, and deposition time), and ambient parameters [39,40]. The release kinetics of the active substance also depends on the way it is incorporated in electrospun mats. Naveen et al. reported the 95% release of kanamycin sulphate-loaded PHB nanofiber mats within the first 8 h when the antibiotic was entrapped on the surface and sandwiched within the nanofiber mats [41]. However, some applications need much slower release of the active substances.

The purpose of this study was (1) to investigate the formation of porous morphologies of electrospun mats in relation to the solvent viscosity, and (2) to assess the resulting drug entrapment efficiency and drug release profile. Levofloxacin was selected as the model drug; it is an antibacterial agent belonging to the group of fluoroquinolones and has good solubility in chloroform. Its antibacterial activity is based on the inhibition of the supercoiling activity of bacterial DNA gyrase and halting DNA replication [42]. The hypothesis was that the porous morphology would support easier incorporation of drug into the PHB matrix and might increase the drug entrapment efficiency. The type of formed morphology should subsequently influence the drug release kinetics. Recently, several works confirmed the biocompatibility of PHB electrospun mats despite the use of chlorinated solvents during processing [43]. Despite all of the aforementioned investigations, there is a lack of information on the influence of different morphologies, other than uniform fibers, on the drug release kinetics of electrospun meshes. Moreover, the addition of bioactive compounds often alters the morphology of electrospun mats [44,45]. In the present work, the viscosity of the electrospinning solvent was modified by the variation in the PHB concentration in the solvent, and by the ratio of dichloromethane and chloroform used as a binary solvent system. The morphology of the developed electrospun mats was proven by scanning electron microscopy and the drug release profile was monitored in vitro in phosphate-buffered saline solution.

## 2. Materials and Methods

### 2.1. Preparation of PHB Electrospun Films

Poly(3-hydroxybutyrate) (PHB) with a weight-average molecular mass (M_w_) of 350 kg·mol^−1^ and polydispersity Đ of 1.2 was obtained from the Nafigate Corporation, Prague, Czech Republic. Scaffolds with various morphologies were produced by electrospinning as follows: PHB was dissolved in dichloromethane/chloroform in the ratio of 1:1 (sample set I), 1:2 (sample set II) and 1:3 (sample set III) at a concentration of 1%, 2%, 4%, 5% and 8% (w/v). PHB scaffolds loaded with levofloxacin (Sigma Aldrich, Saint-Louis, MO, USA) were prepared by the addition of 1 wt.% of the drug (related to the PHB mass) into the dissolved PHB and stirring for 15 min. For the preparation of fiber meshes, the resulting polymer solution was placed into a 10 mL syringe fitted with a metallic needle with a diameter of 1 mm. Electrospinning was performed on a laboratory-made device with two high voltage sources, one positively charged (0–25 kV) and the other negatively charged (−15–0 kV). The positive charge was placed to the tip of the needle through the spinning solution, pushed by a syringe pump. The negative charge was placed on a collector, which was represented by a metallic round plate with a diameter of 10 cm and covered by a flat aluminum sheet. Each sample was collected on the new aluminum sheet. The conditions of the electrospinning process are described in Table 1. The prepared electrospun meshes were left 24 h in a hood and 10 h at 40 °C in a vacuum oven to evaporate solvents. The complete evaporation of solvents was confirmed by thermogravimetric analysis (data are not presented in this work). The electrospun samples without antibiotic were designated as EM_X, where X indicates the concentration of PHB in the solution used for the electrospinning. Additionally, electrospun samples loaded with levofloxacin were designated as EM_X_L.

To determine the concentration of levofloxacin entrapped in PHB electrospun meshes, 100 mg of sample was dissolved in 5 mL of chloroform. One mL of dissolved sample was mixed with 10 mL of sodium phosphate buffer (0.1 mol·L^−1^, pH 7.4) for 30 min. The solution was filtered through 0.45 μm cellulose acetate membrane before UV-VIS determination of levofloxacin. The experiments were run in triplicate. The concentration of levofloxacin in the solution was determined by means of UV-VIS spectrophotometry at 292 nm. Subsequently, the entrapment efficiency was calculated by using the following equation:

(1) $$\mathrm{Entrapment}\;\mathrm{efficiency}\;\left(\%\right)=\frac{\mathrm{Weight}\;\mathrm{of}\;\mathrm{drug}\;\mathrm{in}\;\mathrm{electrospun}\;\mathrm{film}}{\mathrm{Initial}\;\mathrm{weight}\;\mathrm{of}\;\mathrm{drug}\;\mathrm{in}\;\mathrm{solvent}\;\mathrm{for}\;\mathrm{electrospinning}}\times 100$$

### 2.2. Characterization

#### 2.2.1. Determination of Dynamic Viscosity

The dynamic viscosities, η, of the prepared solutions, were determined by using a rotating viscometer (Fungilab Alpha L, Barcelona, Spain) at 20 °C. The standard volume of the samples was 100 mL.

#### 2.2.2. Morphological Analysis

Surface morphology of the PHB fiber meshes was investigated by scanning electron microscopy (FEI Versa3D SEM/FIB, Oregon, OR, USA). The microscope was operated under high-vacuum mode at an acceleration voltage of 5 kV. The surfaces were sputtered with a 5 nm layer of Pt.

#### 2.2.3. Fourier Transform Infrared Spectroscopy (FT-IR)

The chemical structure of the prepared scaffolds was characterized by FT-IR in Attenuated Total Reflection mode with a single-reflection diamond crystal using a Nicolet iS50 spectrometer (ThermoFisher Scientific, Waltham, MA, USA). The structure of the electrospun meshes was measured directly on the surface of the samples. Additionally, films were prepared from about 10 mg of electrospun samples with levofloxacin by dissolution in the ratio 1:150 (electrospun sample/chloroform), followed by evaporation. Spectra were collected as the average of 32 scans in the frequency range 4000–400 cm^−1^ with the resolution of 4 cm^−1^. The spectrum of the clean, dry diamond crystal in the ambient atmosphere (air in the laboratory) was used as the background for FTIR measurement.

### 2.3. Drug in Vitro Release Studies

Levofloxacin loaded in PHB electrospun films of 1 cm^2^ with a thickness of 13 μm (EM_1_L), 14 μm (EM_4_L) and 42 μm (EM_5_L) were poured in 2 mL glass vials with 1.5 mL of 0.1 M PBS (phosphate-buffered saline, pH 7.4) and closed. The vials were stored in the shaker at 37 °C and 180 rpm. Levofloxacin release from PHB sheets was determined with UV-VIS spectrophotometry (S-220 Spectrophotometer, BOECO, Boeckel + Co, Hamburg, Germany) at 292 nm for seven days. The concentration of the released levofloxacin was calculated from the intensity of absorbance. The release data are presented as the average value of three specimens with the standard deviation.

### 2.4. Antimicrobial Tests

The antimicrobial activity of levofloxacin incorporated into PHB samples prepared by electrospinning was tested against gram-positive bacterium *Micrococcus luteus* CCM 1569 (ML), gram-negative bacteria *Serratia marcescens* (SM) CCM 8587 and *Escherichia coli* (EC) CCM 7359. For the antimicrobial testing of electrospun films loaded with the drug, agar diffusion tests were conducted. All used microorganisms were supplied by the Czech Collection of Microorganisms, Masaryk University, Brno, Czech Republic. Soybean Casein Digest Agar (Tryptone Soya Agar, Himedia Laboratories, Mumbai, India) was used as the nutrient agar. The samples were cut from the PHB electrospun structures loaded with levofloxacin. The samples with a predetermined weight were placed onto the surface of the agar plate containing a microorganism suspension at a concentration of 5 × 10^5^ CFU per mL. The agar plates were incubated for 24 h at 37 °C. Each sample was tested in triplicate and the growth inhibition halo is presented as the average value with the standard deviation.

## 3. Results and Discussion

### 3.1. Morphology of PHB Electrospun Meshes

**Figure 1:**
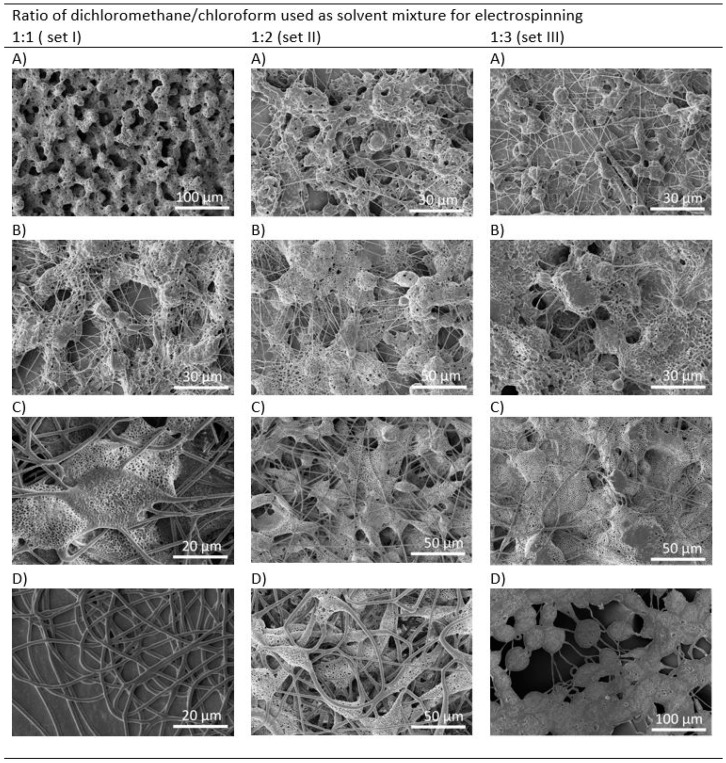
SEM morphology of electrospun meshes processed from dichloromethane/chloroform solution and PHB in the concentration of: (**A**) 1 wt.% (EM_1), (**B**) 2 wt.% (EM_2), (**C**) 4 wt.% (EM_4), and (**D**) 5 wt.% (EM_5).

**Figure 2:**
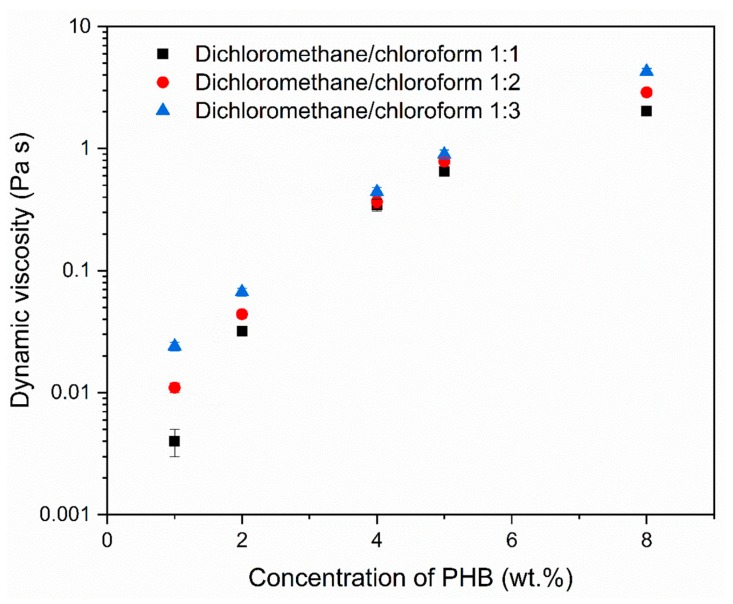
Dynamic viscosity of PHB solution in relation to the ratio of dichloromethane/chloroform (1:1—set I, 1:2—set II, 1:3—set III) and concentration of dissolved PHB.

The concentration of PHB solution was varied from 1 to 5 wt.%. All electrospinning parameters were kept constant; only the composition of binary solvent system was changed. SEM micrographs of electrospun meshes are shown in Figure 1. It is evident from the micrographs that the variation in PHB concentration and ratio of dichloromethane (DCM) and chloroform (CF) in the binary solvent system influenced the formed morphology. All morphologies, except for the model with microfibers, shown in Figure 1D (within set I) are porous. The model shown in Figure 1A (set I) is the only one without fibers and resembles a sponge. Other models contain a mixture of spindle-like units, fibers and grains. The structure of the models formed by using a solvent system with an increased concentration of CF is more fused. Furthermore, the size of the grains increased with the increase in the PHB concentration. One of the objectives was to promote the formation of porous morphology in electrospun meshes. Morphologies within set I with the DCM and CF in the ratio of 1:1 fulfill this criterion. Both solvents, DCM and CF are good solvents for PHB, but have different physical properties (Table 2). Especially, they differ in their evaporation rate. The application of solvent with a higher evaporation rate supports the formation of a porous morphology. Similar findings have been reported by Mahaling and Katti in their work [39]. Providing that all conditions used for electrospinning are stable and only the kind of solvent and PHB concentration are modified, the viscosity of PHB solutions is another parameter that influences the final morphology. Figure 2 shows the increase in the dynamic viscosity with the increased concentration of PHB and chloroform in the solvent. We observed that solutions with PHB in a concentration higher than 5 wt.% partly solidified on the needle and the electrospinning was difficult.

### 3.2. Entrapment Efficiency and Drug Release Kinetics

**Figure 3:**
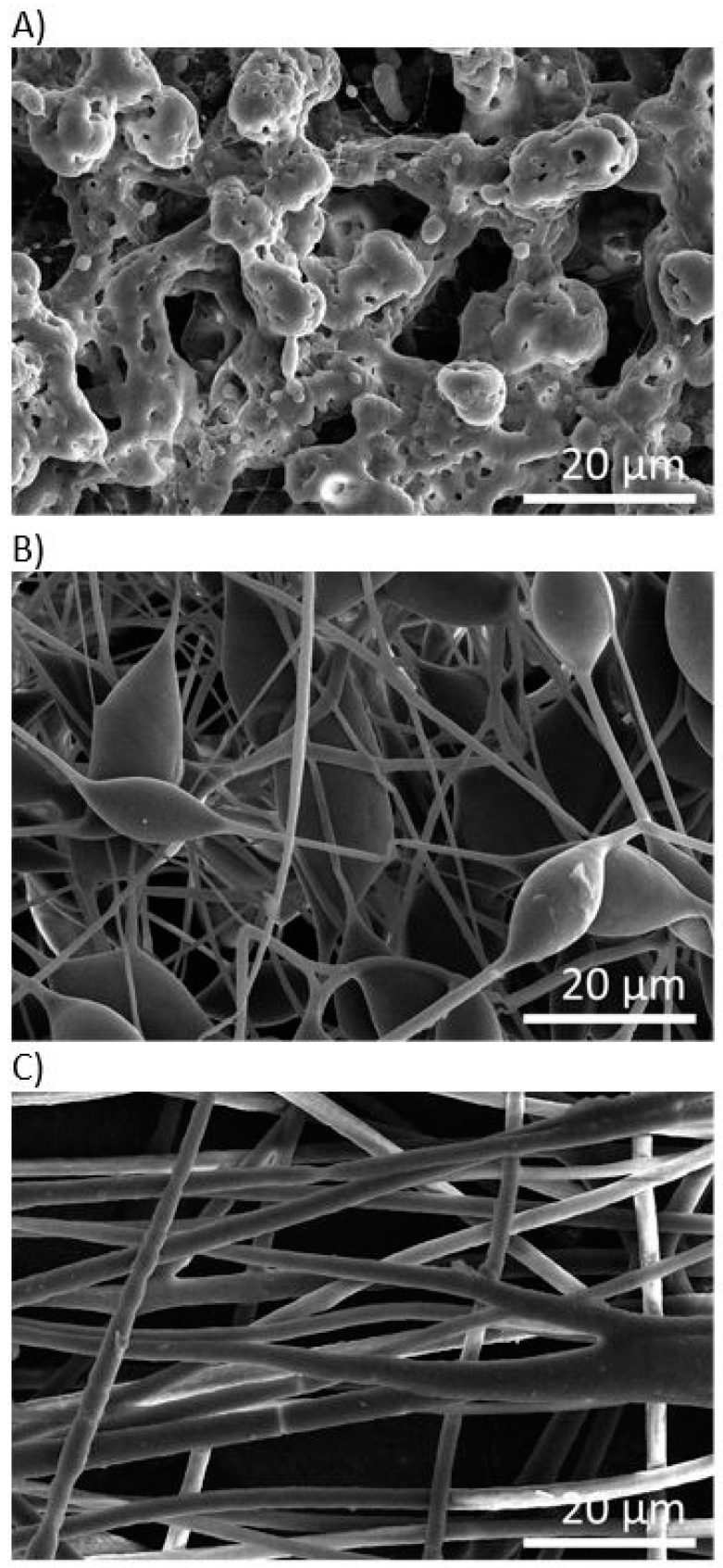
SEM morphologies of PHB electrospun meshes loaded with levofloxacin: (**A**) EM_1_L, (**B**) EM_4_L, (**C**) EM_5_L.

After the morphological screening (see Figure 2), we selected three electrospun models with different morphologies prepared by dissolution of 1, 4 and 5 wt.% of PHB in a binary solvent system, selected from sample set I. The SEM micrographs of the selected electrospun models loaded with levofloxacin are shown in Figure 3. As was hypothesized, the incorporation of the drug influenced the final morphology of scaffolds. In Figure 3A levofloxacin crystals and droplets can be seen on the surface of sample EM_1_L. The original porous structure of the sample EM_4_L disappeared after the incorporation of levofloxacin. Figure 3B,C show fibers with beads and diversified fibers, respectively.

**Figure 4:**
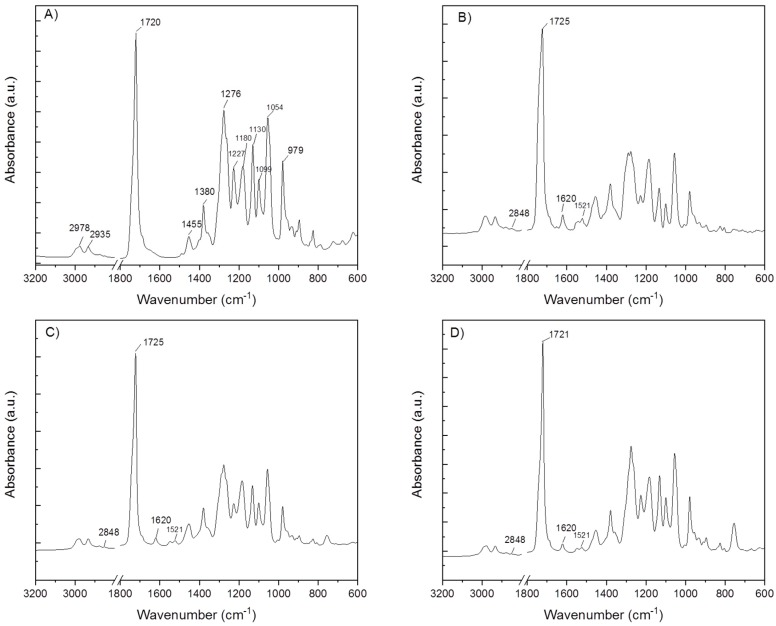
FT-IR spectra of electrospun samples: (**A**) neat PHB, (**B**) EM_1_L, (**C**) EM_4_L, (**D**) EM_5_L.

ATR-FTIR spectroscopic analysis verified the existence of entrapped levofloxacin in PHB electrospun meshes. In Figure 4 the FTIR spectra of the neat PHB electrospun sample and PHB electrospun meshes loaded with levofloxacin are shown. The FTIR spectrum of neat PHB shows characteristic bands typical for poly(3-hydroxybutyrate) [25]. The ATR-spectra collected from the surface of PHB samples loaded with the antibiotic did not show any differences compared to the neat PHB. However, the films prepared from the dissolution of electrospun meshes with the incorporated levofloxacin confirmed characteristic peaks of levofloxacin. This indicates that the drug was not adsorbed on the surface of the electrospun samples, but it was incorporated inside the meshes. Some characteristic bands typical for levofloxacin were overlapped with the peaks of PHB, e.g., the absorption band at 1725 cm^−1^, which is ascribed to symmetric C=O stretching in ester groups. The bands, which were not overlapped were detected at 2848 cm^−1^ (the peak corresponds to the symmetric C–H stretching in methylene groups.), and 1620 cm^−1^ as well as 1521 cm^−1^ (the band can be assigned to C=C stretching vibrations in the aromatic ring of levofloxacin). This indicates that levofloxacin was successfully entrapped in PHB electrospun meshes. The entrapment efficiency (Equation (1)) was found to be 81.8% for EM_1_L, 22.3% for EM_4_L and 14.4% for EM_5_L. Although levofloxacin was fully dissolved in PHB solutions, which were used for electrospinning, the drug entrapment efficiency decreased with the increased viscosity of the PHB solution. The increase in the viscosity might cause higher sedimentation and thus the drug can adhere to the surfaces of the syringe and the needle. Moreover, the electrospinning was done at room temperature and the solubility of PHB in the chlorinated binary solvent system was higher at a temperature of about 60 °C.

**Figure 5:**
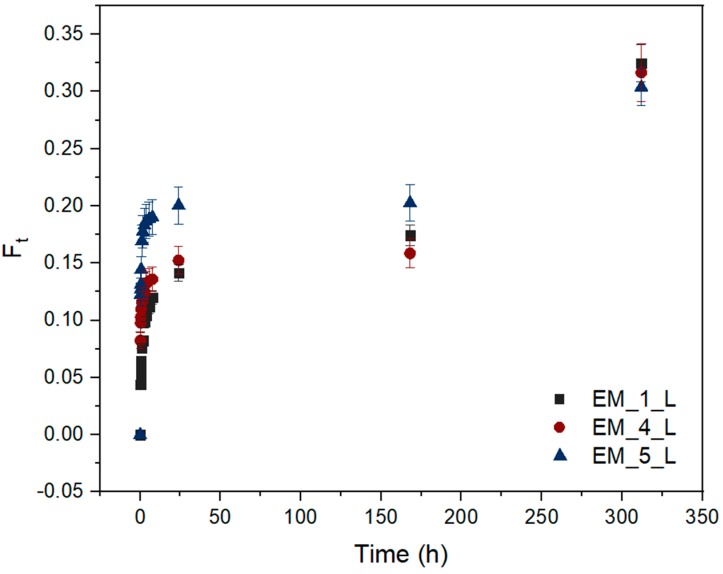
Cumulative fractional release of levofloxacin from electrospun films EM_1_L, EM_4_L and EM_5_L. (F_t_—fraction of drug released in t time. The data points and error bars represent mean values and standard deviation of data sets, respectively).

The levofloxacin release profiles from the three above mentioned electrospun samples were recorded from the immersion in PBS solution (pH 7.4, 37 °C) for 13 days. The solubility of levofloxacin is pH dependent and reaches about 30 mg·mL^−1^ at 20 °C and pH 7.5 [40]. The release of the drug incorporated in a degradable polymer matrix depends on many factors. PHB is a hydrolysable polymer and the course of its degradation in PBS solution relies on its molecular weight, crystallinity and morphology. Moreover, hydrolysis is a time, temperature, and pH dependent process. When all parameters except the morphology of electrospun meshes were kept constant, samples gradually released about 14–20% of the drug during the first 24 h (see Figure 5). The release of the drug began with a slight burst effect after the first 10 min and continued with a gradual release up to 24 h. This release tendency indicates the surface erosion of the polymer matrix. The course of the drug release was changed when the bulk erosion of PHB started. The visible changes in the release tendency were detected after the investigated samples were immersed in PBS solution for 13 days. It is interesting to note that sample EM_1_L, which released only 14% of the entrapped drug after 24 h, overtook the other two models and reached a cumulative drug release of 32.5%. In spite of different morphologies, all the tested electrospun models reached a comparable cumulative drug release of about 30.4–32.5% after 13 days. These findings correspond to the use of the same type of PHB. At the beginning of drug release test, the main differences arose from the different morphologies. The morphology of the electrospun meshes markedly influenced the capability of the sample to entrap the drug as well as the drug release rate during the first stage of degradation, which corresponded with the variations in the surface erosion.

### 3.3. The Antimicrobial Susceptibility of PHB Loaded Levofloxacin Electrospun Meshes

**Figure 6:**
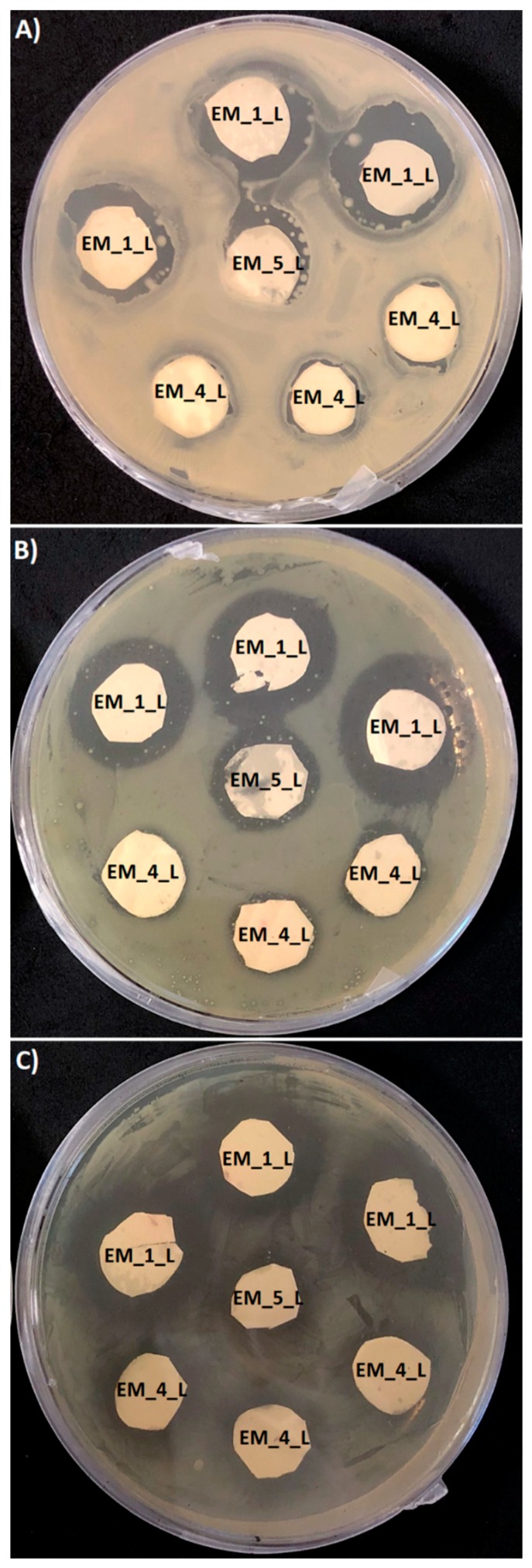
Antimicrobial activity of PHB electrospun meshes M_1_L, M_4_L and M_5_L tested against (**A**) *Micrococcus luteus*, (**B**) *Serratia marcescens*, and (**C**) *Escherichia coli*.

The antimicrobial susceptibility of different electrospun meshes loaded with levofloxacin was determined using the agar diffusion test (Figure 6). The antimicrobial activity of samples with a surface of 1 cm^2^, was recorded after 24 h as a growth inhibition halo. Due to the variation in the samples’ thickness, the determined values were correlated with the corresponding values of the mesh thickness (Table 3). All tested PHB meshes showed distinctive antimicrobial activity against the gram-positive bacterium (*M. luteus*) and gram-negative bacteria (*S. marcescens* and *E. coli*) with the following order of efficiency: EM_1_L > EM_4_L ≥ EM_5_L. The antimicrobial efficiency corresponded with the amount of the entrapped antibiotic and with the morphology of the mesh. It should be highlighted that levofloxacin is an antibacterial agent with a broad spectrum of activity against gram-positive and gram-negative bacteria. Interestingly, the activity of levofloxacin incorporated into PHB electrospun meshes displayed much higher efficiency against the gram-negative bacteria compared to gram-positive bacteria. Gram-positive bacteria compared to gram-negative bacteria are usually more susceptible to the action of antibiotic agents, mainly due to differences in the structure and composition of their cell walls [46,47]. Therefore, these PHB electrospun meshes with levofloxacin seem to be highly promising for tissue engineering applications. They could contribute to the prevention and treatment of wound and surgical site infections caused by gram-negative bacteria [48,49].

## 4. Conclusions

Electrospinning of poly(3-hydroxybutyrate) in a dichloromethane/chloroform binary solvent system was demonstrated as a simple method for the preparation of scaffolds with different morphologies. Systems that resulted in sponge, beaded fibers and fiber morphologies were selected for the incorporation of the drug. Based on its solubility, levofloxacin was selected as the model drug. PHB electrospun scaffolds differed in their entrapment efficiency of the drug. The sponge model was able to entrap about 81.8% of drug. The other two models with a fiber structure with or without beads, were much less effective and entrapped 22.3% and 14.4%, respectively, of the originally loaded drug. All of the electrospun models gradually released from 30.4% to 32.5% of the incorporated levofloxacin after 13 days. When the sample was first immersed in PBS solution, the drug release course was influenced by the surface erosion and later it was also by the bulk erosion. All tested electrospun models entrapped and released a sufficient amount of levofloxacin to exhibit their antimicrobial efficiency. Based on the drug entrapment efficiency and the drug release profiles it can be concluded that the herein proposed electrospinning method of PHB, resulting in EM_1_L film with a sponge morphology might have the potential for the development of an efficient, controllable drug release system. However, faster in vitro hydrolysis of PHB needs to be promoted. This will be a subject of our further research.

## Figures and Tables

**Table 1:** Set up conditions used for electrospinning of PHB solutions.

Electrode Distance (mm)	Syringe Diameter (mm)	Needle Diameter (mm)	Voltage Difference (kV)	Intensity of Electrical Field (kV/mm)	Feed Rate (µL/min)
200 ± 1	18.0	1.0	20.0 ± 0.1	1	100

**Table 2:** Physical properties of solvents.

Solvent	Boiling Point (°C)	Dynamic Viscosity at 20 °C (MPa)	Vapour Pressure at 20 °C (kPa)	Dielectric Constant at 20 °C	Surface Tension at 20 °C (mN·m^−1^)
Dichloromethane	40	0.43	47	9.1	28.1
Chloroform	61	0.58	21	4.8	27.2

**Table 3:** Antimicrobial activity conducted as growth inhibition halos (average value ± standard deviation, n = 3).

Sample	Growth Inhibition Halo (mm)					
	Micrococcus Luteus		Serratia Marcescens		Escherichia Coli	
	^a^ Measured Value	^b^ Corrected Value	Measured Value	Corrected Value	Measured Value	Corrected Value
EM_1_L	3.7 ± 1.1	3.7 ± 1.1	4.1 ± 1.1	4.1 ± 1.1	5.1 ± 1.1	5.1 ± 1.1
EM_4_L	0.8 ± 1.0	0.75 ± 0.9	1.2 ± 1.2	1.1 ± 1.1	2.0 ± 1.1	1.9 ± 1.0
EM_5_L	2.1 ± 1.2	0.65 ± 0.4	2.2 ± 1.1	0.68 ± 0.3	3.0 ± 1.0	0.93 ± 0.3

^a^ measured value—the value of the growth inhibition halo determined on the agar; ^b^ corrected value—the value of the growth inhibition halo corrected according to the thickness of the sample (in relation to sample EM_1_L).
